# Why Is Theory of Mind Important for Referential Communication?

**DOI:** 10.1007/s12144-016-9492-5

**Published:** 2016-08-10

**Authors:** Francesc Sidera, Georgina Perpiñà, Jèssica Serrano, Carles Rostan

**Affiliations:** University of Girona, Facultat d’Educació i Psicologia, 17071 Girona, Spain

**Keywords:** Theory of mind, Referential communication, Cooperation, Children

## Abstract

This research studies the relation between children’s Theory of Mind (ToM) and the communicative behaviour and strategies used in a referential communication task. A total of 46 children (aged 6 to 10) were administered 6 ToM tasks, and they also participated in pairs in a cooperative task. Each pair built 4 construction models. Results showed that several ToM skills were related to the communicative behaviours of requesting clarification and giving information. In addition, the most used communicative strategy was Joint review, in which participants reviewed together the location of their blocks. This strategy was the most related to ToM abilities and to cooperative success. The importance of ToM for developing the communicative behaviours and strategies necessary for cooperation is discussed.

## Introduction

The capacity to understand people’s mental states and use them to predict people’s behaviour (also called theory of mind, or ToM), might have consequences in how we regulate our behaviours in communicative interactions (Resches and Pérez Pereira 2007). In this sense, the aim of this research is to study the relation between theory of mind in children and referential communication. Specifically, our purpose is to study which ToM skills are more related to different communicative behaviours (such as making clarification requests) and strategies that occur in referential communication. We also intend to find out which ToM skills and communicative behaviours or strategies are the most relevant in explaining success in a referential communication cooperative task.

### Development of Referential Communication Skills

Referential communication skills refer to the capacity to transmit verbally to a conversational partner the representation of an object, event or idea that constitutes the benchmark for a message (San Martín et al. 2014). These skills are important for social adaptation. For example, social interaction problems may arise when children have difficulty in verbalizing inadequate messages (Abbeduto and Short-Meyerson 2002; Abbeduto et al. 2008). The ability to communicate with others begins to develop very early, but the ability to select the most appropriate expressions in each situation continues to improve until adolescence (Matthews et al. 2012). In the specific case of referential communication, it seems to be quite developed around the age of six years, and continues to improve until at least the age of 10 (Pynte et al. 1991).

One of the key skills for successful referential communication is to detect the ambiguity of messages. This requires not only understanding what is communicated linguistically, but also to integrate this information with the state of knowledge of the receiver as well as with the communicative context (Nilsen and Graham 2012). Messages cannot be deemed to be appropriate or inappropriate by themselves, but conversational partners need to understand each other within a particular context. For example, over-specifying information about the referent may be a good way to facilitate the identification of the referent (Arts et al. 2011). We will now focus on the skills that are important for referential communication, from the viewpoint of both the speaker and the listener.

#### Referential Communication Skills of the Speaker

Liszkowski et al. (2008) found that, at the age of 12 months, when children communicate with their parents, they are capable of pointing adequately to objects depending on the parent’s prior knowledge about the referent. Once they have acquired language, they also adapt it to the conversational partner. For example, 3-year-old children can modify their speech according to whether their conversational partner can see a referent or not (Campbell et al. 2000). In a similar vein, from the age of 2 children already provide appropriate information when they are asked to clarify an ambiguous message, and they may learn from clarification requests (Matthews et al. 2007). Furthermore, a child’s capacity to learn from clarification requests seems to be higher when clarification requests are specific rather than general, and when the speaker provides information about the cause of their not understanding (Matthews et al. 2012).

Nilsen and Graham (2012) pointed out that one essential aspect of communication capacity relies on the appreciation of communicative ambiguity. As early as in primary school, children may reformulate their messages without the need to receive clarification requests, so they are aware of the potential ambiguity their messages may have for receivers. In a similar way, Nilsen and Graham (2012) observed that 5-year-old children were aware that a message produced by another person could be ambiguous from the viewpoint of the receiver, without even knowing what the right information was. In this respect, from the age of 6 years, children start producing linguistic expressions more appropriate to the viewpoint of the listener, producing sentences that contain enough information to avoid ambiguity (Nilsen and Fecica 2011). However, despite their early capacity for realizing that messages may be ambiguous, children aged from 5 to 7 years still have problems giving helpful information so their conversational partner can solve ambiguities, even when the receiver acknowledges that the referent has not been understood (Karabenick and Miller 1977). In fact, one of the difficulties that referential communication represents for children is perceiving which characteristics of a given referent are the most relevant for communicative acts, or what distinguishes one referent from the others.

#### Referential Communication Skills of the Receiver

Children aged from 4 to 6 years have not yet acquired the ability to assess the ‘informativeness’ of a message (Roby and Kidd 2008). In addition, 5–6 year-olds still have difficulty in distinguishing what a speaker says literally from what the speaker intends to communicate, and it is difficult for them to realize that ambiguous messages may lead to a state of knowledge different from what the speaker had intended (Beal and Belgrad 1990). However, they do have the capacity to differentiate between ambiguous and informative messages if, for example, they are told that the speaker might want to trick them (John et al. 2009). Besides, it seems that school-age children tend to overestimate their own knowledge when interpreting ambiguous messages (Nilsen and Graham 2012).

One of the important skills for understanding messages is informing the speaker about the non-comprehension of the message (Skwerer et al. 2013), as well as the ability to make clarification requests regarding ambiguous messages, an ability that develops with age (Jeanes et al. 2000). However, in relation to the ability to make clarification requests, Skwerer et al. found that it was more important to consider the types of requests rather than their quantity. In summary, it is not until 8 or 9 years of age that children acquire a level of proficiency as receivers almost equal to that of adults (John et al. 2009).

### ToM and Referential Communication Skills

By ToM we refer to the capacity to make inferences on our own and others’ mental states (such as, beliefs, desires or intentions), as well as to predict people’s behaviour based on those inferences. ToM is therefore a fundamental capacity that develops gradually from birth, and it is a crucial cognitive development that has been studied intensively in recent years. It may also be understood as a set of skills with different degrees of complexity that appear throughout development and expand our understanding of human behaviour (Serrat et al. 2013). Several authors have studied the development of ToM in children, finding that, despite individual differences in relation to when these abilities are acquired, typically-developing children follow a similar developmental pattern, so the order of acquisition of these skills seems to be relatively stable (Wellman et al. 2011).

ToM skills enable us to manage situations of social competence (Byrne and Whiten 1988) because they allow us to understand deception, lies or false beliefs about reality; and in humans they enable communication and cooperation with others (Moll and Tomasello 2007). Although many of the studies about ToM have placed special emphasis on deception or Machiavellianism, it is appropriate to stress the importance of ToM skills for the cooperative mind. Indeed, people with low-level mentalistic skills are not prone to collaborating with others or engaging in altruistic behaviour, just as they have difficulty in understanding other people’s deceptions or simulating behaviour (Liebal et al. 2008; Sally and Hill 2005).

In order to communicate efficiently, people must bear in mind the interlocutor’s viewpoint (John et al. 2009). Using it as a guide for our communicative behaviour is an essential component for the capacity to use and interpret appropriately the language used in social interactions. The distinction between the capacity to understand other people’s minds, and using it to guide our communicative behaviour is thus pertinent (Nilsen and Fecica 2011).

Several studies support a close relation between ToM and the capacity to communicate effectively in a cooperative task (Paal and Bereczkei 2007; Resches and Pérez-Pereira 2004; Sidera et al. 2013). Specifically, some referential communication skills have been linked to a child’s capacity to understand (that people may hold) false beliefs, such as: the ability to identify pictorial references in oral messages, to understand directions on a map, to detect and resolve ambiguities in oral messages (John et al. 2009; Maridaki-Kassotaki and Antonopoulou 2011), to use references appropriately in the production of narratives (Charman and Shmueli-Goetz 1998), to use self-regulation (Olivar et al. 2004), or to adapt verbal instructions to the knowledge and needs of the listener (Resches and Pérez-Pereira 2004).

Olivar et al. (2004) have studied the relation of referential communication skills not only with first-order false-belief understanding, but also with second-order false-belief understanding (understanding that people may be wrong when attributing beliefs to other people), and they found that the quality of the message in referential communication tasks was related to the capacity to understand second-order false beliefs. In addition to false-belief understanding, other ToM skills have been associated with referential communication abilities. For example, Roberts and Patterson (1983) identified a strong relation between perspective-taking and referential communication skills. Specifically, they found that referential communication was related not only to the ability to be aware of the information that another person possesses, but also to the ability to identify the specific information that this person needs. Indeed, in order to communicate with another person we need to consider what the other person knows and what he or she needs to know, in order to interpret appropriately a message in a given context.

Apart from considering the state of knowledge of our conversational partners, and the context in which communication occurs, it is also important to look at the intention of their utterances, and to attend to the communicative clues (voice, facial expressions) that provide information about these intentions and allow us to adjust our behaviour and mental states to them (Nilsen and Fecica 2011). In this sense, Happé (1993, 1994) found that children’s performance in batteries of ToM is a good predictor of the understanding of figurative language (irony, metaphor, jokes, etc.) and its underlying motivations.

People with difficulties in developing mentalistic skills show social and communication difficulties (Liebal et al. 2008; Rivière and Nuñez 1996), particularly with referential communication (Dahlgren and Dahlgren Sandberg 2008). However, having better mentalistic skills may not always imply having better referential communication capacities. At least, this is what Krych-Applebaum et al. (2007) suggested from the results of their study. They asked adults to participate in a referential communication task where an instructor had to give verbal instructions to a builder in order to enable the latter to reproduce a construction. They found that high scores in the Reading the Mind in the Eyes task (see Baron-Cohen 2003) in the builders could be counter-productive, and hypothesised that it could lead them to be overconfident about what the instructor wanted to communicate (making incorrect assumptions), or it could lead them to feel bad about asking for information, which would prevent them from making sure they understood the other person.

Understanding that people have false beliefs is useful in communication, because it permits us to consider that the understanding of reality that people create from our information may not correspond to reality, or to our interpretation of reality. However, the fact that we understand that other people may hold false beliefs does not imply that we will always consider other people’s beliefs in our communicative exchanges. Executive function demands (for example, having to consider multiple pieces of information simultaneously), as well as emotional and motivational factors, have an influence on how we use the understanding of false beliefs in our communicative behaviour (Nilsen and Fecica 2011). In fact, some authors suggest a deep dissociation between the ability to distinguish the beliefs of others and how this skill is used to interpret their actions. Even adults often ignore what other people know when interpreting the meaning of their messages (Keysar et al. 2003). Along these lines, some authors point out the difficulty, even in adults, to take into account the knowledge of other people in order to interpret their messages efficiently, leading to egocentric errors (Apperly et al. 2010).

A few authors have focused on studying which communicative strategies children use in referential communicative situations. For example, Johnston et al. (1997) presented a task to preschool children in which one child had to describe to another child two target toys from a group of three. The authors found different strategies for describers; from simpler strategies consisting of describing specific characteristics of individual objects, to more complex strategies, contemplating all objects as a group and defining the common characteristics that differentiated target objects from other objects. We may therefore distinguish two different strategies to communicate a message; a local strategy, which consists of describing the referent, and a global strategy, in which the referent is described in relation to other possible referents, in terms of the characteristics that make it unique (Roby and Kidd 2008).

Johnston et al. (1997) found a cognitive load effect on communication strategies, which could explain why people sometimes use communicative strategies that are less efficient in terms of words (e.g., giving redundant messages), but may be more efficient in terms of the effort and time necessary to formulate them. Thus, different types of communicative strategies may be used to achieve a communicative goal, provided that all the strategies are available in a given task (for example, pointing may be useless if the other person cannot see you). Besides, the fact that children with fewer linguistic skills used complex strategies less often in Johnston et al.‘s study, and the fact that ToM and language development are closely linked (Resches et al. 2010), suggest that ToM may be linked to the choice of different communicative strategies in referential communication tasks. Therefore, in the present study, we also intend to consider how the acquisition of ToM skills is related to the use of the communicative strategies that children use in a referential communication task. Furthermore, we also want to study the effect of communicative strategies on success in a referential communication task. In this respect, the work by Barbieri and Iozzi (2007) is relevant. In a study using a referential communication task with children aged 4 and 5 they found that the use of analogies was an effective strategy for the instructors to direct the attention of the builders toward the referents, having an impact on the effectiveness of the referential communication.

In sum, the objectives of the present paper are: a) to study which ToM skills are more closely related to the communicative behaviour and strategies used in a cooperative task; and b) to study the influence of the communicative behaviour and strategies on the outcome of a referential communication task. In order to achieve these objectives, a set of ToM tasks were administered to a group of children, and a referential communication task was designed in which children needed to cooperate to build together different block constructions. The choice of the ToM tasks was intended to represent a wide range of ToM skills that children acquire during their childhood, from easier to more challenging tasks (Serrano 2012).

## Method

### Participants

A total of 46 children participated in the study. They were divided into 2 groups according to their school level: 24 were first-graders (12 girls and 12 boys) and 22 fifth-graders (10 girls and 12 boys). The ages of the young group ranged between 72 and 83 months (mean age: 78.17 months; SD: 3.24), while the old group ranged from 121 to 131 months (mean age: 127.77 months; SD: 2.54). All participants were recruited from two public schools in the area of Girona (in Spain). The parents of all the children gave written consent for their participation in the study. Apart from these 46 children, data from 2 pairs of fifth-graders were collected, but they were not included in this investigation for problems with video recording.

### Material

Three types of tasks were administered: ToM tasks, the cooperative task and the IQ test. Next we explain them in detail:

#### ToM Tasks

Participants were administered the 6 ToM tasks explained below. All scores were transformed to a 0–3 point scale, so that all tasks had equal importance in the sum of ToM task scores that were calculated.

- First-order false belief

Children’s understanding of first-order false beliefs was assessed using the unexpected content task (Perner et al. 1987). First, participants were shown a closed tube of Smarties® and the experimenter asked them what they thought was inside. After the children answered, the researcher showed the real content of the tube (pebbles), which were introduced again inside the tube. Next, the children were asked several questions. First, they were asked about their initial belief about the contents of the tube, and also a control question about its real content. Afterwards, the participants were asked what a classmate would think was inside the tube when seeing it for the first time; participants were also asked to justify their answer. Finally, participants were asked to respond to another control question to ensure that they knew their classmate had not known the real content of the tube. The two control questions were a prerequisite for obtaining any score. The total score in this task was 2 points (later transformed to 3); one point if children responded correctly to the question regarding their own false belief, and another point if the question about the classmate’s belief was correct.

b. Second-order false belief

In order to assess the children’s second-order false-belief understanding, a task from the Developmental Neuropsychological Assessment II (NEPSY – II; Korkman et al. 2014) was used. This task is a version of the classic ‘change of location’ task. In the first part, participants were introduced to the main characters of the story (Mary and John) and they were shown a coloured picture of a fair. The experimenter explained that John wanted to go on the big wheel while Mary decided to go on the carousel. Following the procedure from the above-mentioned test, the narrator explained that John had finally decided to go to another attraction (the haunted house). Participants were asked about Mary’s belief about John’s location. In the second part of the task, participants were told that Mary had actually seen John going to the second attraction (the haunted house), but John did not know that Mary had seen him. So, children had to predict where John thought Mary would look for him. They were also asked to justify their answer. Finally, participants were asked 2 control questions. The range score varied from 0 to 1. Children who answered correctly all the questions (tests, justification and control questions) were given 1 point (transformed to 3).

c. Deception

A version of the task used by Filippova and Astington (2008) was used to assess the understanding of deception. The narrator explains a story about two siblings. One of them (Marta) never tells the truth, and her brother (Peter) knows that. Then the experimenter explains that one day Peter could not find his soccer ball and he was sure that Marta had hidden it either in the closet or under the bed. Then Peter asked Marta where the ball was, and Marta answered that it was under the bed. At this point, the participants were asked why Peter went to look for the ball in the closet, and a control question was also used. One point (later converted to 3) was given to children responding correctly to both questions.

d. Metaphor

In this task, children were administered the Metaphor task included in NEPSY-II (Korkman et al. 2014). Participants were shown a coloured picture of two twin sisters, and the experimenter explained that their mother said: “they are like two drops of water”. Participants who correctly identified the meaning of this expression were given 1 point (transformed to 3).

e. Faux-pas

Understanding faux-pas situations was assessed by using a version of one of the stories created by Baron-Cohen et al. (1999). The story was explained as follows. Cristina gave a toy plane to Manuel for his birthday. A few months later, they were playing with Manuel’s plane, and Cristina accidentally broke it. She apologized, and Manuel said: “Don’t worry. I never liked it anyway. Someone gave it to me for my birthday”. Afterwards, the children were asked the following questions:

1. “In the story, did anyone say something they shouldn’t have said or something awkward?”
2. “Who said it?”
3. “What did he/she say?”
4. “Did Manuel want to hurt Cristina?”
5. “How did Manuel make Cristina feel”?
6. “What did Cristina give Manuel for his birthday?”
7. “Did Manuel remember Cristina had given him the plane for his birthday”?

Children were given one point if all the questions referred to the identification of the faux-pas were correct (the three first questions). If the first three questions were correct, a second point was awarded if questions 4 and 5 were also correct (about the non-intention to hurt and the feeling caused), provided that control questions 6 and 7 were also correct The maximum score for this task was 2 points (then transformed to 3).

f. Emotion attribution

The subtest called ‘Contextual task’ from the NEPSY-II (Korkman et al. 2014) was used to assess the ability to identify the correct emotion of a character in 7 social situations (1 trial situation and 6 test situations). Participants were shown 7 black and white pictures in which the face of the character (a girl called Julia) was not shown. The child was then asked to identify the correct facial affect from four possible photographs. The maximum score was 6 points; 1 point was given for responding correctly to each situation (that score was then converted to 3 points).

#### Cooperative Task

A version of a cooperation task from the study by Krych-Applebaum et al. (2007) was used in the present investigation. In this task, children worked in pairs with classmates to build a model constructed with blocks of Lego Duplo®. In each pair, one child (the guide) gave verbal instructions to another child (the builder). In the first-grade group there were a total of 12 couples: 6 of the same sex (3 boy-boy and 3 girl-girl) and 6 of opposite sex. In the fifth-grade group, there were 11 pairs: 5 of the same sex (3 boy-boy and 2 girl-girl) and 6 of opposite sex. Each pair of children built a total of 4 construction models: one 4-block trial model and three 6-block test models of progressive complexity. The first test model only contained pieces that varied in colour and size, but not in shape; furthermore, all the pieces were located on the board, using an x and y axis. Both the second and third model varied in colour, size and shape, but while the second model had only one figure that was on top of another, the third model had 5 figures that were located on top of other figures. These models were designed following a pilot study.

The children sat facing each other and an opaque screen was placed between them in order not to receive visual information about the workspace. Children were explained that they could talk but not look at the others’ workplace, and that no help on the part of the researcher would be offered. After explaining the objective of the task (to build together a replica of the guide’s model), the experimenter gave a model to the guide and 45 construction blocks to the builder. These blocks were different in four dimensions: colour, size, shape and position along the coordinate axis. In the first part of the task, children were asked to construct the trial model and when they finished, the opaque screen between them was removed so they could see their result and discuss how they had built it. In the second part of the cooperation task, they were asked to construct 3 test models. In this case, they were able to see the final model they had built, but were not permitted to discuss it.

For each model, a score ranging from 0 to 6 points was awarded (0.5 points if all colours were correct; 0.5 points if all shapes and sizes were correct; 0.5 points if the location of the first piece was correct; 0.5 points if the location of the second piece was correct; and 1 point if the location of each of the 4 remaining pieces were correct, or 0.5 points if the piece was located wrongly only because the location of the previous piece was wrong). The maximum score was 18 points (‘Cooperation Score’), because the construction of the trial model was not taken into account in this score.

### IQ

The Kaufman Brief Intelligence Test (K-BIT) by Kaufman & Kaufman and Kaufman (1994) was used to assess children’s Intelligence Quotient. This is a test consisting on a verbal and a nonverbal scale. From the verbal scale, 6-year-olds were administered the expressive vocabulary task and 10-year-olds both the definition and the expressive vocabulary task. Also, the nonverbal matrices task was administered to both groups. For data analyses, we used the percentile scores of the IQ composite score, a measure including the verbal and the nonverbal scores.

### Procedure

Data were collected in 2 sessions. In the first session ToM and IQ were assessed. Children were tested individually in a quiet room at their schools, and the administration of tasks was identical for each child. The first session lasted between 30 and 45 min.

The second session was carried out one week after the first one. In this session, children performed the Cooperative task, which was recorded by video camera. The experimenter explained to the participants that their aim was to do their best in constructing together a replica of different block models. They were also informed that they could not look at their partner’s working area. So, the main objective of this investigation was to build the models as similarly as possible to the models designed by the experimenters.

#### Total ToM Score

As explained in the instruments section, the children’s score in each of the ToM tasks was transformed to a range of 0–3. Moreover, a Total ToM score was calculated by adding the scores of the 6 ToM tasks. Hence, the Total ToM score had a possible range from 0 to 18 points.

#### Communicative Behaviour Classification and Scoring

The communicative behaviour of the participants was divided into 2 groups of categories. On the one hand, behaviours related to asking for clarification and giving information. On the other hand, the participants’ communicative strategies and mistakes were analysed. We will now explain all the categories considered in each of the groups.

### Clarification Requests and Information-Giving Behaviour

Here we describe the categories used to analyse the behaviour related to asking for clarification about the information given by the conversational partner, and related to giving information to the listener.

#### General Clarification Requests (CR)

This category includes all participants’ clarification demands that do not refer to any specific aspect of the Lego® pieces. In other words, this category includes general questions that the children asked their conversational partner that do not mention specific information about colour, size, shape, or the position of a piece. This category includes expressions such as: “I don’t understand”, “What?” or “Can you repeat?”

#### Colour CR

Includes clarification requests about the colour of the piece “What’s its colour”?

#### Shape CR

Includes questions about the shape or the size of the piece, like “Do you mean the longest one, made of six buttons?”

#### Position-General CR

This category refers to the clarification requests that refer to the position of the pieces, but do not ask for specific information regarding that position. For example: “Where?”

#### Position-Referent CR

When the children formulated clarification requests aimed at clarifying which piece is the referent of the conversational partner’s explanation. For example, if the speaker says “Put it to the right”, the builder might say “To the right of the orange piece, or to the right of the blue piece?”

#### Position-Right/Left CR

When the participants’ clarification requests were aimed at clarifying whether a specific piece was supposed to be placed to the right or to the left in relation to another piece. For example, if a builder says: “Take a blue piece and put it next to the red piece”, the builder might say “To the right or to the left of the red piece?”

#### Position-Vertical/Horizontal CR

When questions are addressed at clarifying the vertical/horizontal orientation of the piece of construction. For example: “Is the piece facing forward or to the side?”

#### Position Front/back CR

When questions are made to clarify whether a piece is placed in front or behind another piece. Example: “Does the blue piece go in front of the red piece?”

#### Position up/down CR

Includes clarification requests aimed at clarifying whether another piece should be located on top, or below, another piece. “Is the red piece located on top of the yellow one?”

#### Giving information about the location of the pieces (only for builders)

Included here were all utterances by builders aimed at giving information to the guide about the position of his/her pieces. For instance, if the builder says: “I have the blue one with two buttons on the left of the red one”.

#### Asking information about the location of the pieces (only for guides)

Includes questions made by the guides directed at asking the builder about the location of his/her pieces. For example, if the guide asks: “Do you have the blue one next to the right one?”

One video was created for each construction model. Therefore, there were 4 videos for each pair of children. Two raters (two of the authors of the article) watched each of the videos. In each model, the raters classified the behaviour of the participants in each of the previous categories, according to whether they had displayed that behaviour (1 point was given in this case), or not, in that model. Therefore, each pair of children obtained a score from 0 to 4 in each of the above-described categories. For example, 0 points if they had not displayed that behaviour in any of the 4 models, 1 point if they had displayed that behaviour in one of the models, etc. Hence, the maximum score was 4 in each category. When disagreements occurred, both raters watched the video together and reached an agreement by discussion.

### Communicative Strategies

The following categories were used for the analysis of communication strategies (and communication mistakes) during the cooperation task:

#### Use of Platform

This strategy consists of turning the platform on which the pieces are built in order to imagine how the other person has, or should have, his or her pieces.

#### Turn of Head or Body

In this case the guide or the builder turns their head or body in order to imagine how the other has, or should have, his or her pieces.

#### Use of External Spatial Referents

Consists of using an external spatial referent to communicate the position of the pieces. For example: “Put it to the side of where your wall is”.

#### Use of Geometric Figures

In this category we included all sentences that used shapes (like geometric figures) in order to communicate the position/location of the pieces. For example: “The pieces should look like a bridge”.

#### Proposal of Common Ground

When one of the children suggested a shared referent to use in future communications. For example: “When I say right, I will always refer to *my* right”.

#### Visual Perspective-Taking Mistakes

Here we considered communicative mistakes consisting of giving some information without considering that the other person cannot see you, your pieces, or your working area, and thus, cannot understand your message. For example, saying “Put it there” while pointing to one of your pieces, without realizing that the other person cannot know which one is being referred to.

#### *Individual relocation* (only for Builders)

When the builder changes the location of some pieces (relocation) that had already been located without informing the conversational partner.

#### Joint Review

This strategy consists of talking about the location of the pieces in order to ensure that they are well placed.

As in the case of communicative strategies and mistakes, in each of the four construction models, two of the article’s authors categorised whether the guide and the builder had used the above-mentioned strategies or not. Again, if the raters disagreed with the categorisation, they watched the video together and found an agreement through discussion. In each construction model both the guide and the builder were awarded either a “yes” or a “no” in each of the categories. They were awarded one point if they had a yes. The score of the 4 models was added up, so each child obtained a score ranging from 0 to 4 in each of the categories.

## Results

All the analyses described in the results section were carried out with IBM SPSS version 19.

### Inter-Rater Reliability

In the case of clarification requests and information-giving/asking behaviours, a total of 1840 behaviours were classified as occurring or not occurring within a particular construction model. The percentage of agreement between the two raters was 93.91 %, and Cohen’s Kappa was 0.80.

In the case of communicative strategies, a total of 1288 responses were classified as using or not using a particular communicative strategy within a particular construction model. The percentage of agreement between the two raters was 95.73 %, and Cohen’s Kappa was 0.65.

### Results on IQ

Scores on the IQ test were used to make sure that the guides and builders had similar IQ scores. Using the Mann-Whitney test, we found no differences between them (*U* = 191.500; *p* = .108). Similarly, when we compared the IQ scores of guides and builders in each grade, we found no differences neither in first- (*U* = 43.500; *p* = .099) nor in fifth-graders (*U* = 49.500; *p* = .470).

### Results Describing ToM Abilities

In Table 1, an improvement from first- to fifth-graders may be observed in some ToM tasks. In general, there is an important change in the performance of ToM tasks according to the age of the participants, so a clear progression exists in the Total ToM score from the younger to the older group. Focusing on each of the tasks, we observed a significant improvement in the following tasks: Second-order false belief, Faux-pas and Emotion attribution. In the case of the Deception and Metaphor tasks, there was a considerable difference between younger and older children, but it was not significant in either case. Differences were minor in the case of 1st-order false belief.

**Table 1:** Mean (and SD) of ToM tasks according to children’s school grade

		1st-order FB	2nd-order FB	Deception	Metaphor	Faux-pas	Emotion Attribution	Total ToM score
1st grade	*N = 24*	2.75	.75	2.12	1.75	1.44	1.92	10.73
		(.57)	(1.33)	(1.39)	(1.51)	(1.36)	(.50)	(2.85)
5th grade	*N = 22*	2.86	2.86	2.73	2.45	2.32	2.52	15.75
		(.44)	(.64)	(0.88)	(1.18)	(1.20)	(.50)	(2.95)
1st Vs 5th	*U*	244.0	78.0^b^	211.0	202.0	171.0^a^	108.0^b^	43.5^b^
	*p*	.451	.000	.090	.087	.023	.000	.000

The maximum score in all tasks is 3, except in the Total ToM score (18). “*U*″ informs that the test used was Mann-Whitney’s U. The same is applicable to the following tables. Superscript letters indicate significant differences between groups, ‘a’ at the *p* < .05 level, and ‘b’ at the* p* < .01 level

### Results Describing Communicative Behaviour

Table 2 shows the results of the communicative process regarding clarification requests and information-giving/asking behaviours, by role and school grade. We observed some significant differences according to the grade. Specifically, we found improvements in the Position-vertical/horizontal CR category. Taking into account the role of participants, we observed that 5th grade builders displayed less General CR, more Vertical-horizontal CR and more Giving information about the location of the pieces. Chi-square analyses showed that in the last two categories there were more fifth-graders than first-graders that exhibited these behaviours at least once during all the models (Vertical-horizontal CR: *χ2* (1, *N* = 23) = 7.340, *p* = .007; Giving information: *χ2* (1, N = 23) = 5.316, *p* = .021). In the case of guides, clarification requests were rarely made, and no age differences were observed.

**Table 2:** Mean (and SD) of clarification requests and information-giving/asking behaviours according to children’s role and school grade

		Gen CR	Colour CR	Shape CR	Pos Gen CR	Pos Ref CR	Pos R/L CR	Pos V/H CR	Pos F/B CR	Pos U/D CR	GI	AI
1st grade	*Guides*	.42	.08	.00	.08	.00	.00	.00	.00	.08		.25
	*N = 12*	(.515)	(.289)	(.000)	(.289)	(.000)	(.000)	(.000)	(.000)	(.289)		(.452)
	*Builders*	1.83	1.67	2.17	1.92	1.25	1.17	.17	.42	1.25	.25	
	*N = 12*	(1.12)	(1.23)	(1.19)	(1.31)	(1.29)	(1.27)	(.39)	(.67)	(1.60)	(.62)	
5th grade	*Guides*	.36	.00	.27	.09	.09	.00	.00	.00	.00		.73
	*N = 11*	(.505)	(.000)	(.467)	(.302)	(.302)	(.000)	(.000)	(.000)	(.000)		(.101)
	*Builders*	.91	1.91	2.45	1.00	2.09	1.91	1.00	.82	1.73	1.36	
	*N = 11*	(.70)	(1.04)	(.93)	(1.41)	(1.45)	(1.45)	(.78)	(.87)	(1.19)	(1.29)	
1st Vs 5th in guides	*U*	62.5	60.50	48.0	65.5	60.0	66.0	66.0	66.0	60.5		49.5
	*p*	.833	.740	.288	.976	.740	1.00	1.00	1.00	.740		.227
1st Vs 5th in builders	*U*	31.0^a^	56.5	58.0	42.0	43.0	46.0	26.0^a^	49.0	49.0	32.0^a^	
	*p*	.032	.566	.651	.151	.169	.235	.013	.316	.316	.037	
1st Vs 5th (all)	*U*	202.0	256.5	226.5	220.0	215.0	228.5	187.0^a^	233.0	233.5		
	*p*	.146	.856	.382	.254	.225	.357	.019	.344	.429		

The possible range in all the columns is 0–4. CR = Clarification request; Gen = General; Pos Gen = Position-general; Pos ref. = Position-referent; Pos R/L = Position Right/Left; Pos V/H = Position Vertical/Horizontal; Pos F/B = Position Front/Back; Pos U/D = Position Up/Down; GI = Giving information about the location of the pieces; AI = Asking information about the location of the pieces. Superscripted ‘a’ indicates that *p* < .05

### Results Describing the Communicative Strategies

Table 3 shows the results of the communication process in relation to the communicative strategies and mistakes. We observed that the most used strategies were: a) in the case of first-grade guides, firstly, the Use of external spatial referents, and secondly, the Use of geometric figures; b) fifth-grade guides used mostly the Joint review strategy, followed by Use of geometric figures and Use of external spatial referents; c) first-grade builders used hardly any strategy; d) fifth-grade builders used the Joint review strategy in first place, and Use of external spatial referents in second place.

**Table 3:** Mean (and SD) of communicative strategies and mistakes according to children’s role and school grade

		Use of platform	Turn of head or body	Use of ESR	Use of GF	Proposal of CG	Visual P-T mistakes	Joint review	IR
1st grade	*Guides*	0	0	.42	.25	0	.33	.08	
	*N = 12*			(.515)	(.452)		(.492)	(.282)	
	*Builders*	0	0	0	.08	0	.08	.08	.58
	*N = 12*				(.289)		(.289)	(.282)	(.996)
5th grade	*Guides*	.09	.27	.36	.64	.09	.09	1	
	*N = 11*	(.302)	(.467)	(.505)	(.505)	(.302)	(.302)	(1.069	
	*Builders*	0	0	.55	.27	.09	.18	1	.27
	*N = 11*			(1.036)	(.467)	(.302)	(.405)	(1.069)	(.467)
1st Vs 5th (guides)	*U*	60.0	48.0	65.0	38.0	60.0	48.5	33.5^a^	
	*p*	.740	.288	.976	.091	.740	.288	.044	
1st Vs 5th (builders)	*U*	66.00	66.00	48.00	53.50	60.00	59.50	33.50^a^	59.00
	*p*	1.0	1.0	.288	.449	.740	.695	.044	.695
1st Vs 5th (all)	*U*	252.0	228.0	230.0	186.0^a^	240.0	240.5	134.0^b^	
	*p*	.296	.064	.332	.035	.135	.433	.000	

The possible range in all the columns goes from 0 to 4. Use of ESR = Use of external spatial referent; Use of GF = Use of geometric figures; Proposal of CG = Proposal of common ground; Visual P-T mistakes = Visual Perspective-Taking mistakes; IR = Individual relocation. *Superscript letters* indicate significant differences between groups, ‘a’ at the* p* < .05 level, and ‘b’ at the *p* < .01 level

Regarding differences between school grades, we observed a significant difference between first- and fifth-graders in the use of the Joint review strategy and in the Use of geometric figures (the latter observed only in builders), so these strategies were used by older children more often. Chi-square analyses showed that in the case of the Joint review strategy, there were more fifth-graders than first-graders using this strategy at least once in all the four models (*χ2* (1, *N* = 46) = 11.578, *p* = .001). Concerning communication mistakes, no significant age differences were observed.

### Relation between Builders’ ToM, Clarification Requests and Giving Information Behaviour

In Table 4, four out of the six ToM tasks were observed to correlate significantly with Giving information behaviour or clarification requests (these, and all the correlations in this study, are Spearman correlations). In particular, the Faux-pas and the Second-order false-belief tasks were the ones that correlated with more elements of communicative behaviour, but the Deception and Emotion attribution tasks also had some significant correlations. In particular, the Deception task correlated significantly and negatively with the Position-general CR, and positively with the Giving information behaviour. However, neither the Metaphor nor the First-order false-belief tasks correlated with the communicative behaviours of Giving information or with clarification requests (of any kind). In addition, the Total ToM score correlated significantly with most displays of Giving information behaviour or clarification requests.

**Table 4:** Spearman correlations between the builders’ ToM and their clarification request and Giving information behaviours

		Gen CR	Color CR	Shape CR	Pos Gen CR	PosRef CR	Pos R/L CR	Pos V/H CR	Pos F/B CR	Pos U/D CR	GI
1st-order	*r*	-.020	-.091	-.277	-.163	.100	.170	.000	.120	.232	-.022
FB	*p*	.926	.678	.200	.457	.648	.437	1.000	.586	.288	.920
2nd-order	*r*	-.212	.399	.567^b^	.085	.555^b^	.429^a^	.529^b^	.391	.403	.444^a^
FB	*p*	.332	.059	.005	.701	.006	.041	.009	.065	.057	.034
Deception	*r*	.000	-.070	.008	-.423^a^	.100	.200	.336	.184	.162	.460^a^
	*p*	1.000	.751	.972	.044	.649	.361	.117	.401	.460	.027
Metaphor	*r*	.087	.014	.196	.072	.213	-.099	.310	.324	.071	.251
	*p*	.693	.948	.370	.743	.329	.652	.151	.132	.747	.248
Faux-pas	*r*	-.110	.478^a^	.222	.131	.578^b^	.426^a^	.734^b^	.470^a^	.598^b^	.486^a^
	*p*	.618	.021	.309	.551	.004	.043	.000	.024	.003	.019
Emotion Attribution	*r*	-.314	.065	.027	-.142	.321	.195	.475^a^	.307	.280	.521^a^
	*p*	.145	.769	.902	.518	.162	.373	.022	.154	.195	.011
Total ToM score	*r*	-.145	.292	.292	-.133	.530^b^	.387	.740^b^	.494^a^	.459^a^	.641^b^
	*p*	.510	.177	.176	.546	.009	.068	.000	.017	.028	.001

CR = Clarification request; Gen = General; Pos Gen = Position-general; Pos ref. = Position-referent; Pos R/L = Position Right/Left; Pos V/H = Position Vertical/Horizontal; Pos F/B = Position Front/Back; Pos U/D = Position Up/Down; GI = Giving information about the location of the pieces. *Superscript letters* indicate significant correlations, ‘a’ at the *p* < .05 level, and ‘b’ at the *p* < .01 level

Focusing on Giving information behaviour and clarification requests, each of them correlated to at least one of the ToM tasks, with the exception of General CR. Furthermore, the General-position CR had a significant correlation with the Deception task, but in a negative direction.

Correlations were also carried out by separating children according to their grade. In the 1st-grade group, the following significant correlations were observed: a) between the Second-order false-belief task and Shape CR (*r* = .697; *p* = .012); b) between the Deception task and Colour CR (*r* = −.580; *p* = .048); c) between the Faux-pas task and Position-referent CR (*r* = .703; *p* = .011) , and between the Faux-pas task and Front/back CR (*r* = .808; *p* = .001); d) between the Total ToM score and General CR (*r* = .646; *p* = .023). In the 5th-grade group, we observed the following significant correlations: a) between the Deception task and Colour CR (*r* = .693; *p* = .018) and between the Deception task and Up/Down CR (*r* = .707; *p* = .015); b) between the Metaphor task and Front/back CR (*r* = .622; *p* = .041), and the Metaphor task and Giving information about the location of the pieces (*r* = .704; *p* = .016); c) between the Faux-pas task and Colour CR (*r* = .619; *p* = .042); d) between the Total ToM score and the Position-vertical/horizontal CR (*r* = .616; *p* = .044).

### Relation between the Guides’ ToM, Clarification Requests, and Asking for Information Behaviour

No significant correlation was found between the guides’ ToM and clarification requests or Asking for information about the location of the pieces behaviour. When correlations were made by school grade, significant correlations were observed only in the group of first-graders, where a negative correlation was observed between the Emotion attribution task and General CR (*r* = −.621; *p* = .031).

### Relation between the Builders’ ToM and their Communicative Strategies

The following significant correlations were observed between the builders’ ToM and the communicative strategies they used: a) between the Use of geometric figures and the Total ToM score (*r* = .426; *p* = .043); between the Joint review strategy and the Second-order false belief (*r* = .521; p = .011), Faux-pas (*r* = .438; *p* = .036), Emotion attribution (*r* = .494; *p* = .017), and Total ToM score (*r* = .657; *p* = .001) tasks. When correlations were made separating the children by school grade, significant correlations were only found in the group of fifth-graders, which were the following: a) between Visual perspective-taking mistakes and the Faux-pas task (*r* = −.714; *p* = .014); b) between the Joint review strategy and the Metaphor task (*r* = .617; *p* = .043).

### Relation between the Guides’ ToM and their Communicative Strategies

When the relations between the guides’ ToM and their communicative strategies and mistakes were studied, a significant correlation was found between the Second-order false-belief task and the use of the Joint review strategy (*r* = .415; *p* = .049). In addition, a negative correlation, very close to significance, was found between the Second-order false-belief task and Visual perspective-taking mistakes (*r* = −.413; *p* = .050). These mistakes also correlated negatively with the Metaphor task (*r* = −.622; *p* = .002) and with the Total ToM score (*r* = −.484; *p* = .019). When correlations were made by separating children according to their school grade, no significant correlations were observed in the first-grade group between the guides’ ToM and their communicative strategies or mistakes. However, in the group of fifth-graders, a negative correlation between the Metaphor task and Visual perspective-taking mistakes (*r* = 1.000).

### Relation between the Score in the Cooperative Task and Communicative Behaviours and Strategies

Correlations were studied between all variables involving communicative behaviours (clarification requests, behaviours related to Giving/Asking for information) and strategies (and mistakes) and the score in the Cooperative task. It was found that this score correlated with the following communicative behaviours: Position-referent CR (*r* = .304; *p* = .040); Position-vertical/horizontal CR (*r* = .439; *p* = .002); Position-front/back CR (*r* = .306; *p* = .039); Position-up/down CR (*r* = .316; *p* = .032). Also, the score in the Cooperative task correlated with the following communicative strategies: Use of geometric figures (*r* = .341; *p* = .020); Joint review (*r* = .616; *p* = .000); and Individual relocation (*r* = −.440; *p* = .036).

A linear regression was also carried out in order to study which of the variables were the best score predictors of the Cooperative task. Thus, the dependent variable was the Cooperation Score, and the independent variables were all the variables that had been considered in the study in relation to communicative behaviour and communicative strategies (and mistakes), as well as the age of the participants in months. The linear regression (see Table 5) was carried out using the ‘stepwise’ method. Three models were obtained; the third one had the best adjusted R square. In this model, the best predictors of the Cooperative Score were first, the Position-vertical/horizontal CR, second the Joint review, and third, the Individual relocation (this one with a negative correlation).

**Table 5:** Forward stepwise multiple linear regression with Cooperation Score as dependant variable, and all variables related to communicative behaviour, strategies and mistakes, and age, as independent variables

Model	Variables in the model	B	Standard error	Beta	t	Sig	F	R Squared	Adjusted Squared R	VIF
1	Constant	4.302	.711		6.048	.000	26.172^b^	.555	.534	1.00
	Position-V/H CR	4.004	.783	.745	5.116	.000				
2	Constant	3.955	.591		6.689	.000	25.195^b^	.716	.687	
	Position-V/H CR	2.752	.741	.512	3.715	.001				1.34
	Joint Review	2.021	.600	.464	3.367	.003				1.34
3	Constant	4.717	.612		7.709	.000				
	Position-V/H CR	2.484	.670	.462	3.705	.002	23.083^b^	.785	.751	1.37
	Joint Review	1.964	.537	.451	3.659	.002				1.34
	Individual relocation	-1.334	.541	-.269	2.465	.023				1.048

Superscripted ‘b’ indicates that* p* < .01

## Discussion

The first objective of this paper was to explore which ToM skills are more related to the communicative behaviour and strategies that children use in a cooperative task. We found that several ToM skills (especially second-order false-belief reasoning and faux-pas understanding) were strongly related to the communicative behaviours and strategies that children use in a referential communication task. What these relations are will be discussed in detail.

The second objective was to analyse the influence of communicative behaviour and strategies in the outcome of a communication process (Cooperation Score). In this respect, it was found that making clarification requests related to the vertical-horizontal axis (on the part of the builder), reviewing together the location of the pieces (Joint review), and not relocating pieces without informing the guide (not using Individual relocation), were the most important communicative strategies for success in the Cooperative task.

Before analysing the relation between ToM skills and the communicative behaviours and strategies in our study, the results obtained in these variables will be reviewed separately. An increase in ToM skills between first- and fifth-graders was found, though it was only significant in the tasks of Second-order false-belief reasoning, Faux-pas, Emotion attribution and the Total ToM score (see Table 1). These results are in accordance with previous research showing an important development in ToM skills between the ages of 6 and 10 (Serrano 2012). In relation to communicative behaviour, differences due to age in the case of the guides were not found. In the case of builders’ communicative behaviour, we found that General CR decreased from 1st-to 5th-graders, whereas Position-vertical/horizontal CR, and the behaviour of Giving information about the location of the pieces increased (see Table 2). Skwerer et al. (2013) highlighted the importance of the type of clarification requests children use. In this respect, we found that both young and old builders used all the strategies, but more children in the older group used certain behaviours (like Position-V/H CR) and to a higher degree. In the case of communicative strategies, a clear age group increase was observed in the Use of geometric figures to describe the position of the pieces, and also in the use of the Joint review strategy (see Table 3). The latter was the most used strategy by fifth-graders, while the most used strategies by first-graders were Use of external spatial referents (guides) and Individual relocation of the pieces (builders). Overall, these results show important age and role differences in the communicative behaviours and strategies used by children in our cooperative task. This suggests that when studying the ToM skills involved in cooperative tasks, the demands of the role of the child (or their age) cannot be disregarded, because they influence the communicative needs imposed on them, and thus, how they actually behave. As previously seen in this type of tasks, the builders’ ToM skills could be more relevant to success in a cooperation activity than the ToM skills of the guides (Sidera et al. 2013).

### Builders’ ToM and Communicative Behaviour

As shown in Table 4, almost all the ToM skills assessed in our study correlate significantly with certain behaviours related to requesting clarification about a given message, or giving information about the location of the model under construction. Only the First-order false-belief and the Metaphor tasks were unrelated in the whole sample with these behaviours, (though the Metaphor task did have a significant correlation with Position-front/back CR in the group of fifth-graders). These results highlight the importance of understanding other people’s mental states in their communicative behaviour, and specifically, for asking questions referring to the communicative intention of the other person (making clarification requests), and for giving information (in the case of the builders too) about the arrangement of the pieces.

In Table 4, we observed that the ToM tasks most related to the different communicative behaviours in the builders were the Second-order false-belief and the Faux-pas tasks. These results are consistent with, and extend, those from Olivar et al. (2004), who found that second-order ToM skills predicted the quality of a message in children. Despite the fact that their study considered different types of communicative abilities, whereas ours was focused mainly on clarification requests, our results show that not only understanding second-order false beliefs are involved in referential communication, but also understanding faux-pas, deception, emotion attribution and metaphor. Therefore, it might be argued that improving all these ToM skills could have an impact on the capacity to communicate about referents in children. This issue will be discussed later on. As suggested already, ToM skills are important, and enable humans to communicate and cooperate with each other (Moll and Tomasello 2007).

As previously mentioned, no relations were found between the understanding of first-order false-beliefs and communicative behaviour. This could be explained by a roof effect, as almost all the children responded correctly to this task, even in the youngest group. Therefore, our results are not in contradiction with previous studies that suggest a relation between understanding first-order false beliefs and referential communication skills (e.g., John et al. 2009; Maridaki-Kassotaki and Antonopoulou 2011; Resches and Pérez-Pereira 2004). Thus, it needs to be considered that different ToM skills might be relevant for communicative behaviour depending on the developmental stage of the child. As children develop more complex ToM skills, these are possibly incorporated to their communicative behaviour. For example, we found that the Metaphor task was related to referential communicative behaviour only in the group of fifth-graders. One possible explanation for this weak relation between metaphor understanding and communicative behaviour could be due to the characteristics of the Cooperative task in our study. This task consisted of discussing different concrete (non-abstract) referents (characteristics of colour, shape and location of different pieces). Hence, it is possible that the capacity to understand people’s metaphorical utterances is more relevant for communicating referentially about more abstract issues. Future research could address this issue, as well as why some ToM tasks were more related to communicative behaviour than others, which raises the issue of how understanding different aspects of our mind changes our communicative behaviour about referents.

When we look at the relations between ToM and the communicative behaviour in builders by considering the two age groups in our study, interesting issues can be found. First of all, in both groups several ToM tasks were related to different types of clarification requests. However, some correlations were age-specific. For example, only in first-graders was a correlation between General CR and the Total ToM score found (see Table 4). Interestingly, we found a significant decrease in the General CR (in builders), and a (non-significant) reduction in Position-general CR between first- and fifth-graders (see Table 2), whereas more specific strategies tended to increase with age, even significantly (Position-vertical/horizontal CR). These results are in agreement with those of Pynte et al. (1991), who suggest that the capacity to make specific clarification requests is acquired around the age of 6, and improved until the age of 10. Indeed, our results suggest that with age children tend to use more specific rather than general clarification requests, but also that ToM skills are related to communicative behaviours differently at different ages: in young children, general CR might be an indicator of high-level ToM skills, whereas in older children it might be an indicator of low-level ToM skills. For example, we found that General CR correlated with the Total ToM score at the age of 6, and not at the age of 10. One possible explanation in terms of mutual understanding is that, in the case of ambiguous messages, it is better to ask something general rather than asking nothing. So making General CR might be a good strategy for young children, because they might start to be aware that messages may be misunderstood, but they might still struggle with monitoring the possible origins or causes of conversational breakdowns (see, for example: Karabenick and Miller 1977). However, with age children develop their communicative skills, and learn to make more specific clarification requests, so General CR are no longer related to high-level ToM skills, and are even related to low-level skills. In this respect, it was found, for instance, that Position-general CR correlated negatively with the Deception task. The latter explanation seems to work well in the case of the builders. However, in the case of the guides, we found a negative relation between General CR and the task of Emotion attribution. So, in this case, General CR was related to low-level ToM skills even in young children. This might be explained by the fact that only a few General CR were made by the guides. It could also suggest that showing certain communicative behaviours could have different meanings depending on the role of the participant in a communicative task.

Again, in relation to age-group differences, it is worth mentioning that the builders’ ToM skills (Total ToM score) were linked to Position-vertical/horizontal CR in the group of fifth-graders, but not in the group of first-graders. In fact, Position-vertical/horizontal CR were almost unused in the group of first-graders. This suggests that in order to communicate efficiently, ToM skills are needed, but it also requires the concepts involved in the task that may act as a source of ambiguity (for example, the vertical-horizontal axis) to be understood and considered.

### Guides’ ToM and Communicative Behaviour

No relations were found between the guides’ communicative behaviour and their ToM skills (apart from the abovementioned negative relation between the Emotion attribution task and the use of General CR in first-graders). This could be due in part to the fact that, when considering their communicative behaviour, mainly clarification requests were analysed (rarely used by the guides, as they were the ones who had privileged information), and perhaps more subtle behaviours that were important for the construction of the common ground were disregarded. We know that when adults are asked to audiotape instructions for the builder, the latter commits more mistakes (Clark and Krych 2004). However, this may be due either to the fact that the guide cannot monitor the understanding of the builder, or because the builder cannot inform the guide about his understanding. Future studies should allow us to disentangle which behaviours of the guide may contribute to monitoring the understanding of the listener, and find out which ToM skills are involved in them. In a previous study, it was found that ToM skills were not important for the result of a referential communication cooperative task (Sidera et al. 2013). As the final decision about the location of the pieces remains in the hands of the builder, it seems logical that their ToM skills contribute to a higher degree to the success of the cooperative task, but it may be the case that with other types of communicative tasks, the ToM skills of the guide are more relevant.

### ToM Skills and Communicative Strategies

We found that the communicative strategy most related to ToM skills was the Joint review, which showed correlations with several ToM tasks, both in builders and in guides. As we will discuss later the Joint review strategy also had an impact on success in the Cooperative task. On the other hand, the Use of geometric figures also correlated with the Total ToM score in builders, and we found that Visual perspective-taking mistakes were negatively related to different ToM tasks in both builders and guides, especially in fifth-graders. Therefore, children who displayed visual communicative behaviours without considering that the other person was unable to see them (e.g., pointing to a specific piece and referring to it) were found to have lower ToM scores. Keysar et al. (2003) found that even adults, despite knowing whether another person has or not some specific knowledge, sometimes do not use that knowledge in interactive situations (for example, considering as a referent for the other person an object whose presence is in fact unknown to that person). Similarly, in our study, even fifth-graders committed Visual-perspective mistakes. More research is needed to study whether the presence of these types of mistakes in children can be used as markers of ToM difficulties. Besides, there is also the need to clarify how communicative behaviours and ToM skills influence at each other in different developmental points, in order to think about future educational interventions aimed at increasing children’s cooperative abilities.

### Relation between the Score in the Cooperative Task and Communicative Behaviours and Strategies

Our second objective was to study the contribution of the communicative behaviour and strategies considered in our study to success in the Cooperative task. In this regard, we found several position clarification request behaviours (especially Position-vertical/horizontal CR) were related to success in the Cooperation Score. The fact that position clarification requests were related to this success, whereas Colour or Shape CR were not, could be due to the fact that in the final score in the Cooperative task, the position of the pieces had a higher weight compared to their colour or shape. On the other hand, the conceptual complexity involved in communicating the position of the pieces was also higher.

Regarding communicative strategies, we found that the Use of geometric figures, the avoidance of Individual relocation, and especially Joint review, were the variables that had a greater impact on success in the Cooperative task. At a research level, this finding highlights the need to understand how ToM skills are involved in the joint construction of a shared system of referents (or common ground). At an educational level, attention should be paid to helping children learn to monitor and review their construction of referents with other people (in cooperative tasks), and understand how to use their understanding of people’s mental states in this process. As our results suggest, a more egocentric understanding of mental states may lead to using strategies that do not consider the Cooperative task as something that should be done together, as in the case of the Individual relocation strategy, which has an effect on success in the Cooperative task. Moreover, in relation to the Use of geometric figures, our findings are in accordance with Barbieri and Iozzi (2007), who found that analogies have a pragmatic function that improves the effectiveness of referential communication. Therefore, our findings recommend that educational work in the field of referential communication should focus on trying to improve children’s abilities to use analogies as a way of sharing, and communication complex referents in the process of constructing a common ground. Understanding how ToM skills and the use of these types of strategies are connected could probably help in this work.

One of the most important limitations of the present study is that, when considering the behaviours and strategies of the participants, we mostly focused on individual rather than on shared behaviours and strategies (with the exception of the Joint review strategy). The children’s proposals for sharing a common ground were taken into account, but only their explicit proposals for sharing an explicit meaning for a word (e.g., “if I say in front, I mean near the screen”) were categorised, and this behaviour was hardly ever observed. However, children may not need these referential pacts to be stated specifically (see Köymen et al. 2014), so more subtle (non-explicit) referential pacts may not have been grasped. Furthermore, the categorisation used in this study did not allow us to analyse the co-construction process of a shared representation of the figures the children were meant to build. This process can only be studied properly if observational tools or categories enable us to observe how two minds work together to understand each other. For example, assessing whether children give appropriate instructions should only be done by considering the whole communicative act, as the speaker may expect the listener to ask questions and arrive in this way at a shared representation of the block they are referring to. Therefore, evaluating whether children give too much or too little information about a block is only possible if we consider their common ground and the evolution of their conversation (see, for example, Arts et al. 2011). Future research should try to ensure that natural situations are created that permit the shared knowledge and implicit pacts of the participants to be controlled at the same time. It should also consider all the possible referents of their utterances, and create categories that not only contemplate the independent utterances of the speaker and listener, but also their interactive communicative behaviour.

In sum, our study suggests that ToM skills and referential communication behaviour (requesting clarification, and giving information about a construction process) are closely related, though this relation differs according to the role and age of the child. ToM skills are also related to communication strategies that involve reviewing together the previously done cooperative work, which is important for success in cooperative tasks. Thus, understanding other people’s minds may allow us to communicate with others more efficiently, avoiding egocentric strategies and sharing our knowledge in a way that permits us to understand each other’s intentions. Future work should focus on studying further how ToM skills guide or influence our way of communicating referentially with others and creating shared common grounds. This would help us to develop educational programs aimed at promoting peer cooperation, which take the understanding of other people’s minds (knowledge, emotions, etc.) as a starting point.
